# Supplementary material for: High cyclic GMP-AMP synthase and stimulator of interferon genes in cholangiocarcinoma suggest their potential as targets for treatment
Source: PeerJ. 2025 Aug 6;13:e19800. doi: 10.7717/peerj.19800 (PMC12335239; doi:10.7717/peerj.19800)
Supplement: Supplemental Information 1 — Kaplan-Meier survival analysis. [file peerj-13-19800-s001.pptx]

## Slide 1
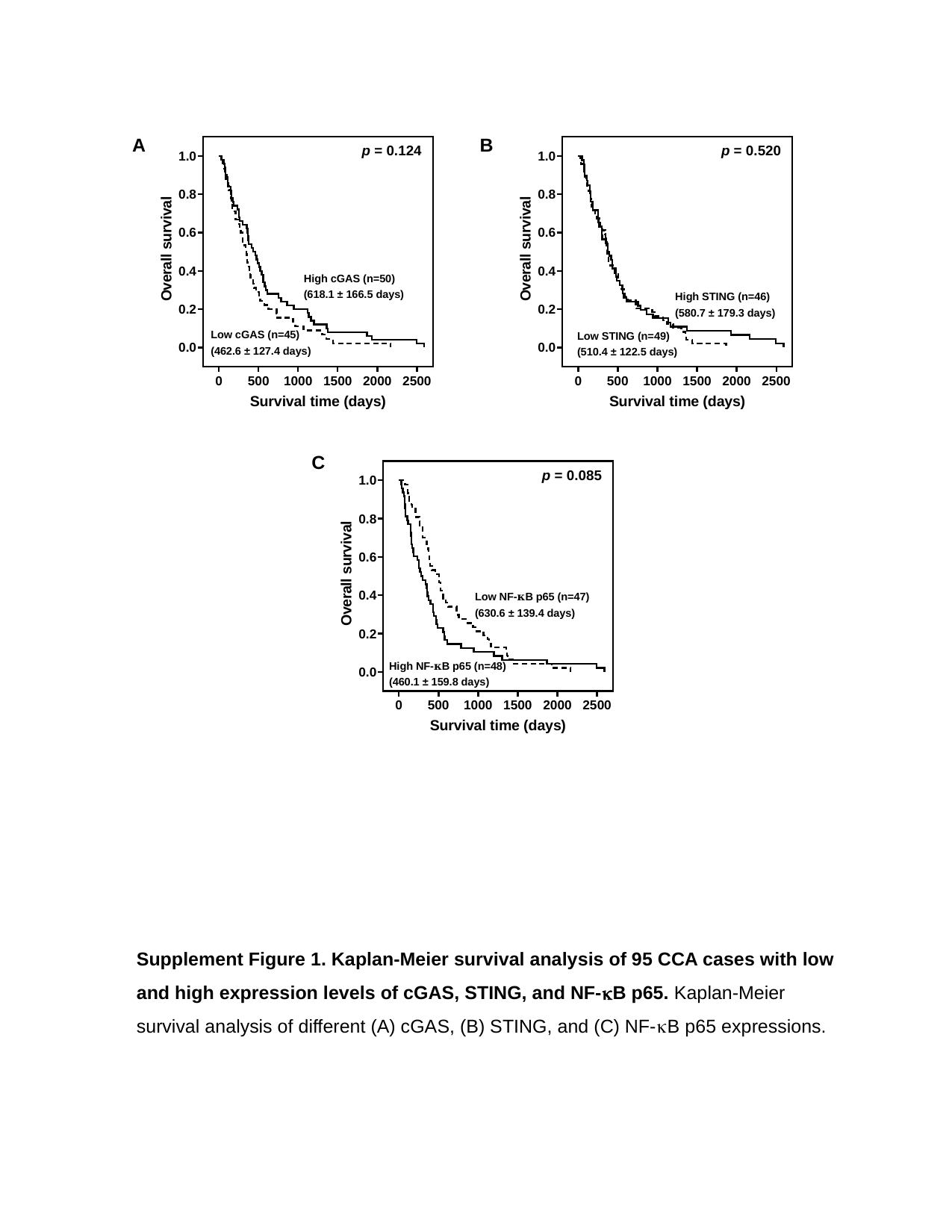

A
p = 0.124
High cGAS (n=50)
(618.1 ± 166.5 days)
Low cGAS (n=45)
(462.6 ± 127.4 days)
B
p = 0.520
High STING (n=46)
(580.7 ± 179.3 days)
Low STING (n=49)
(510.4 ± 122.5 days)
C
p = 0.085
Low NF-𝛋B p65 (n=47)
(630.6 ± 139.4 days)
High NF-𝛋B p65 (n=48)
(460.1 ± 159.8 days)
Supplement Figure 1. Kaplan-Meier survival analysis of 95 CCA cases with low and high expression levels of cGAS, STING, and NF-κB p65. Kaplan-Meier survival analysis of different (A) cGAS, (B) STING, and (C) NF-κB p65 expressions.

## Slide 2
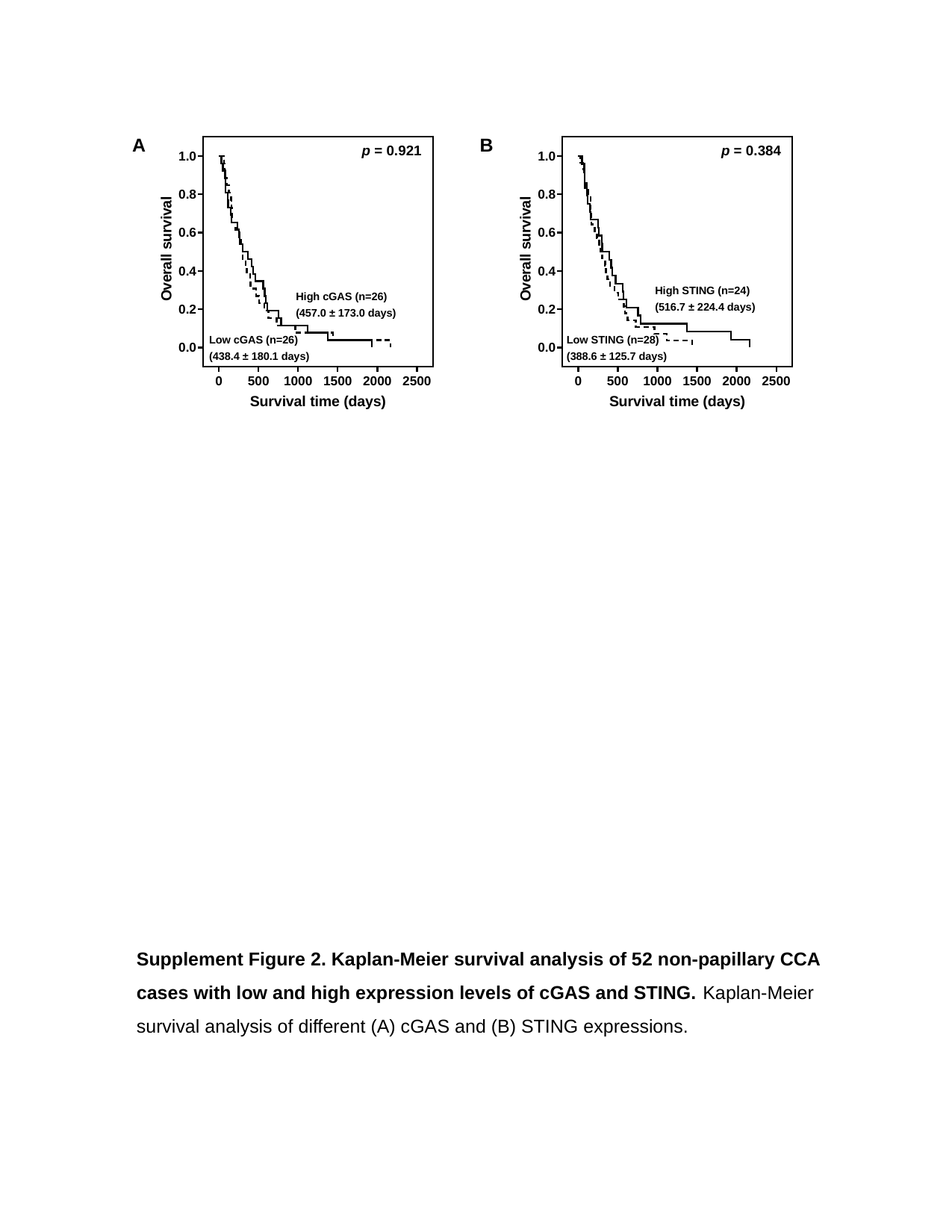

A
p = 0.921
High cGAS (n=26)
(457.0 ± 173.0 days)
Low cGAS (n=26)
(438.4 ± 180.1 days)
B
p = 0.384
High STING (n=24)
(516.7 ± 224.4 days)
Low STING (n=28)
(388.6 ± 125.7 days)
Supplement Figure 2. Kaplan-Meier survival analysis of 52 non-papillary CCA cases with low and high expression levels of cGAS and STING. Kaplan-Meier survival analysis of different (A) cGAS and (B) STING expressions.

## Slide 3
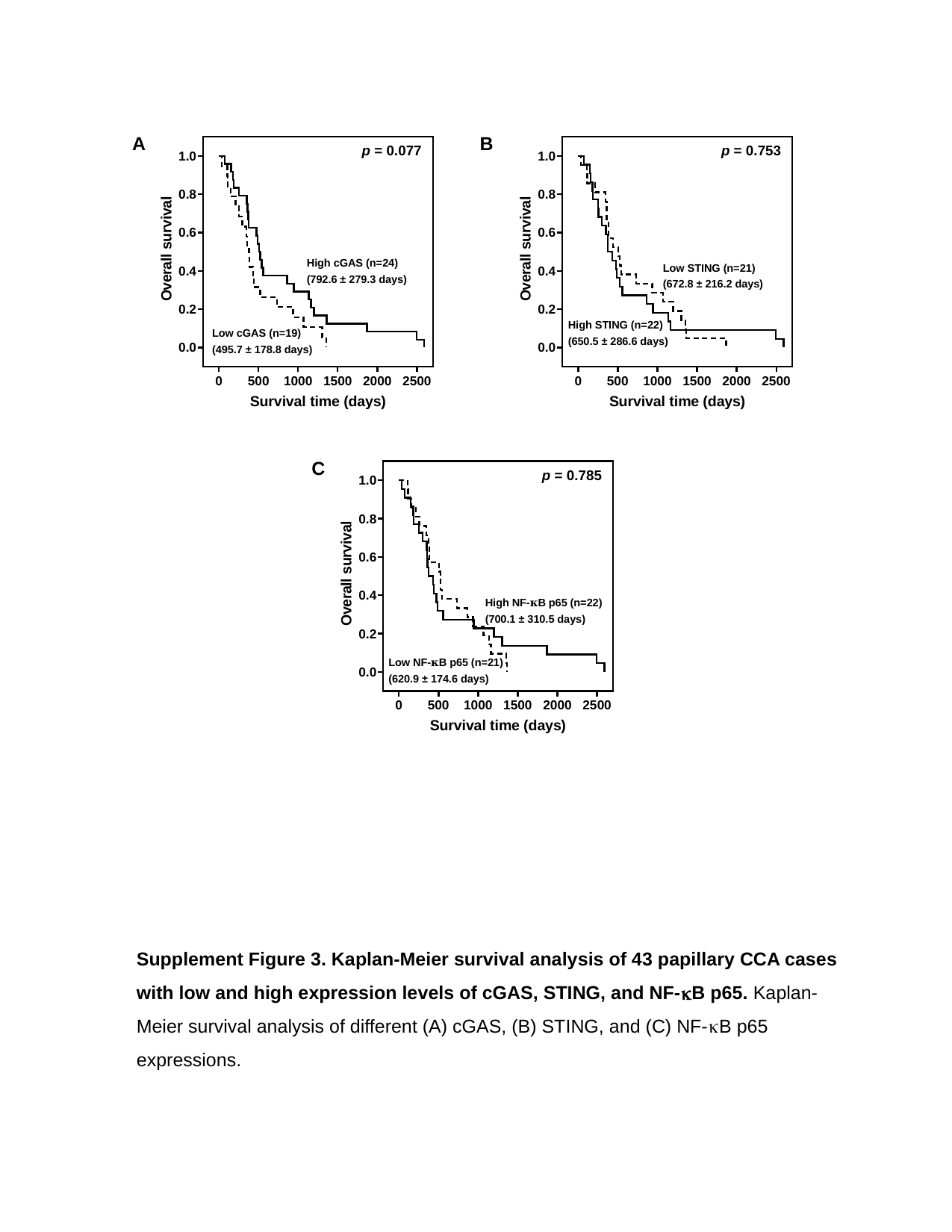

A
p = 0.077
High cGAS (n=24)
(792.6 ± 279.3 days)
Low cGAS (n=19)
(495.7 ± 178.8 days)
B
p = 0.753
Low STING (n=21)
(672.8 ± 216.2 days)
High STING (n=22)
(650.5 ± 286.6 days)
C
p = 0.785
High NF-𝛋B p65 (n=22)
(700.1 ± 310.5 days)
Low NF-𝛋B p65 (n=21)
(620.9 ± 174.6 days)
Supplement Figure 3. Kaplan-Meier survival analysis of 43 papillary CCA cases with low and high expression levels of cGAS, STING, and NF-κB p65. Kaplan-Meier survival analysis of different (A) cGAS, (B) STING, and (C) NF-κB p65 expressions.

## Slide 4
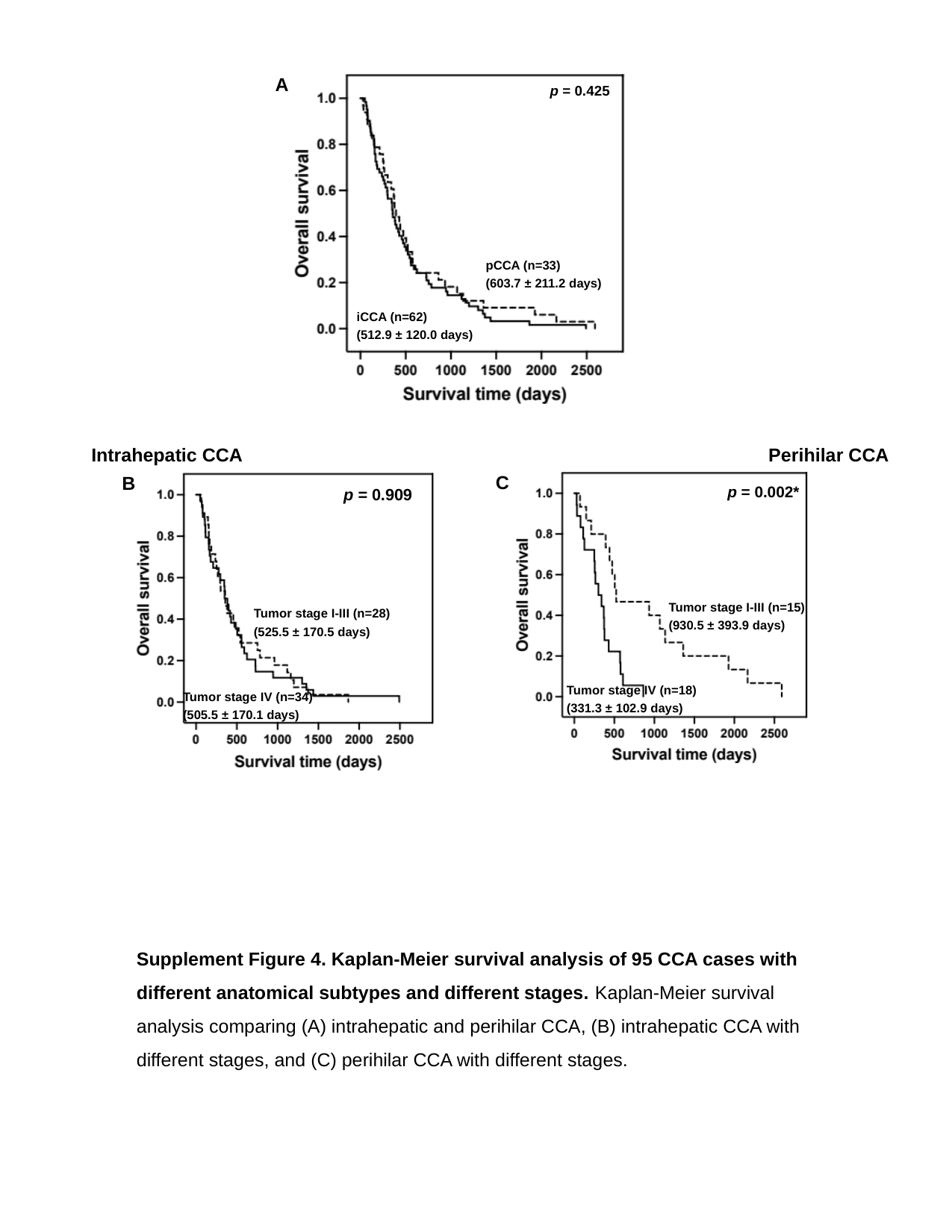

p = 0.425
pCCA (n=33)
(603.7 ± 211.2 days)
iCCA (n=62)
(512.9 ± 120.0 days)
A
Intrahepatic CCA					 Perihilar CCA
C
p = 0.002*
Tumor stage I-III (n=15)
(930.5 ± 393.9 days)
Tumor stage IV (n=18)
(331.3 ± 102.9 days)
B
p = 0.909
Tumor stage I-III (n=28)
(525.5 ± 170.5 days)
Tumor stage IV (n=34)
(505.5 ± 170.1 days)
Supplement Figure 4. Kaplan-Meier survival analysis of 95 CCA cases with different anatomical subtypes and different stages. Kaplan-Meier survival analysis comparing (A) intrahepatic and perihilar CCA, (B) intrahepatic CCA with different stages, and (C) perihilar CCA with different stages.

## Slide 5
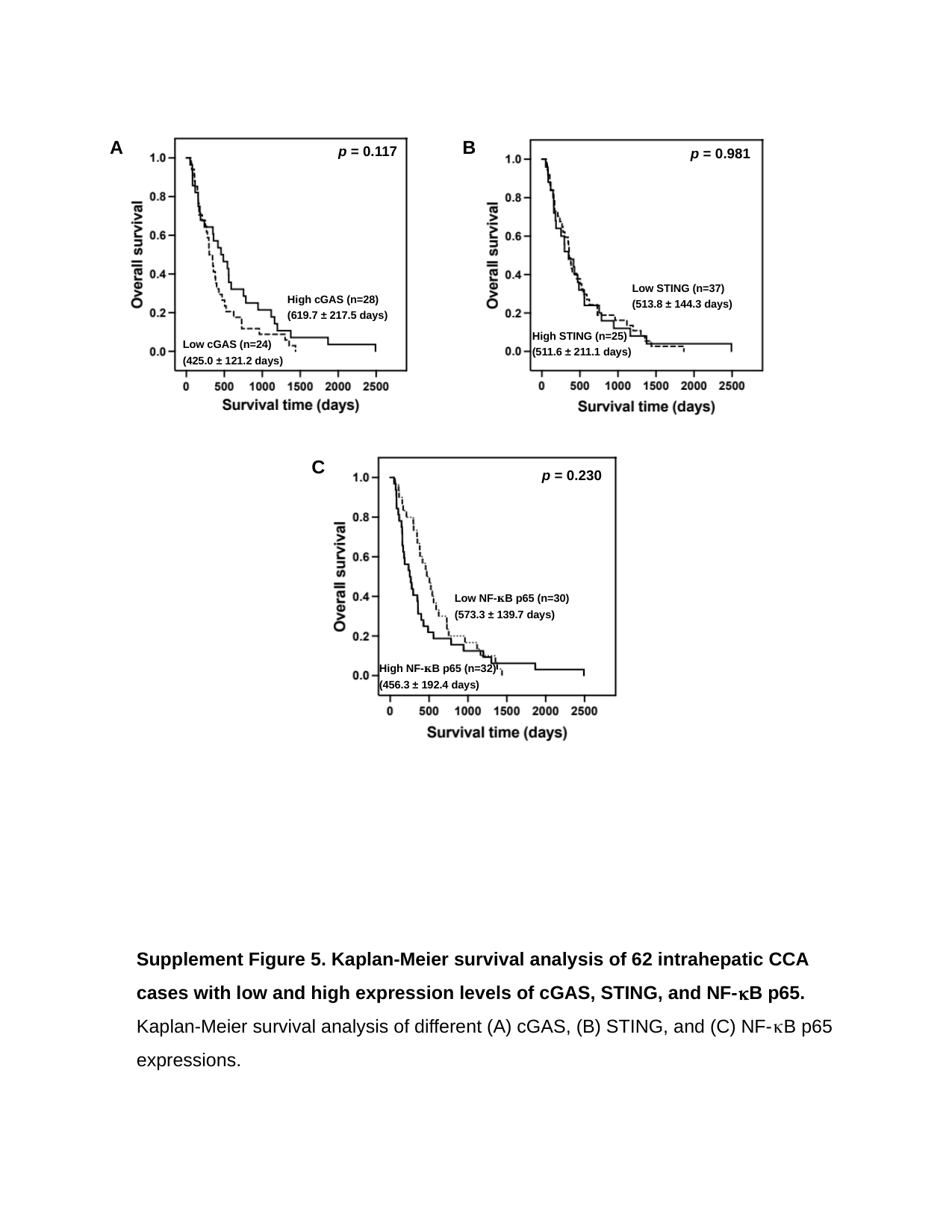

p = 0.981
Low STING (n=37)
(513.8 ± 144.3 days)
High STING (n=25)
(511.6 ± 211.1 days)
A
B
p = 0.117
High cGAS (n=28)
(619.7 ± 217.5 days)
Low cGAS (n=24)
(425.0 ± 121.2 days)
C
p = 0.230
Low NF-𝛋B p65 (n=30)
(573.3 ± 139.7 days)
High NF-𝛋B p65 (n=32)
(456.3 ± 192.4 days)
Supplement Figure 5. Kaplan-Meier survival analysis of 62 intrahepatic CCA cases with low and high expression levels of cGAS, STING, and NF-κB p65. Kaplan-Meier survival analysis of different (A) cGAS, (B) STING, and (C) NF-κB p65 expressions.

## Slide 6
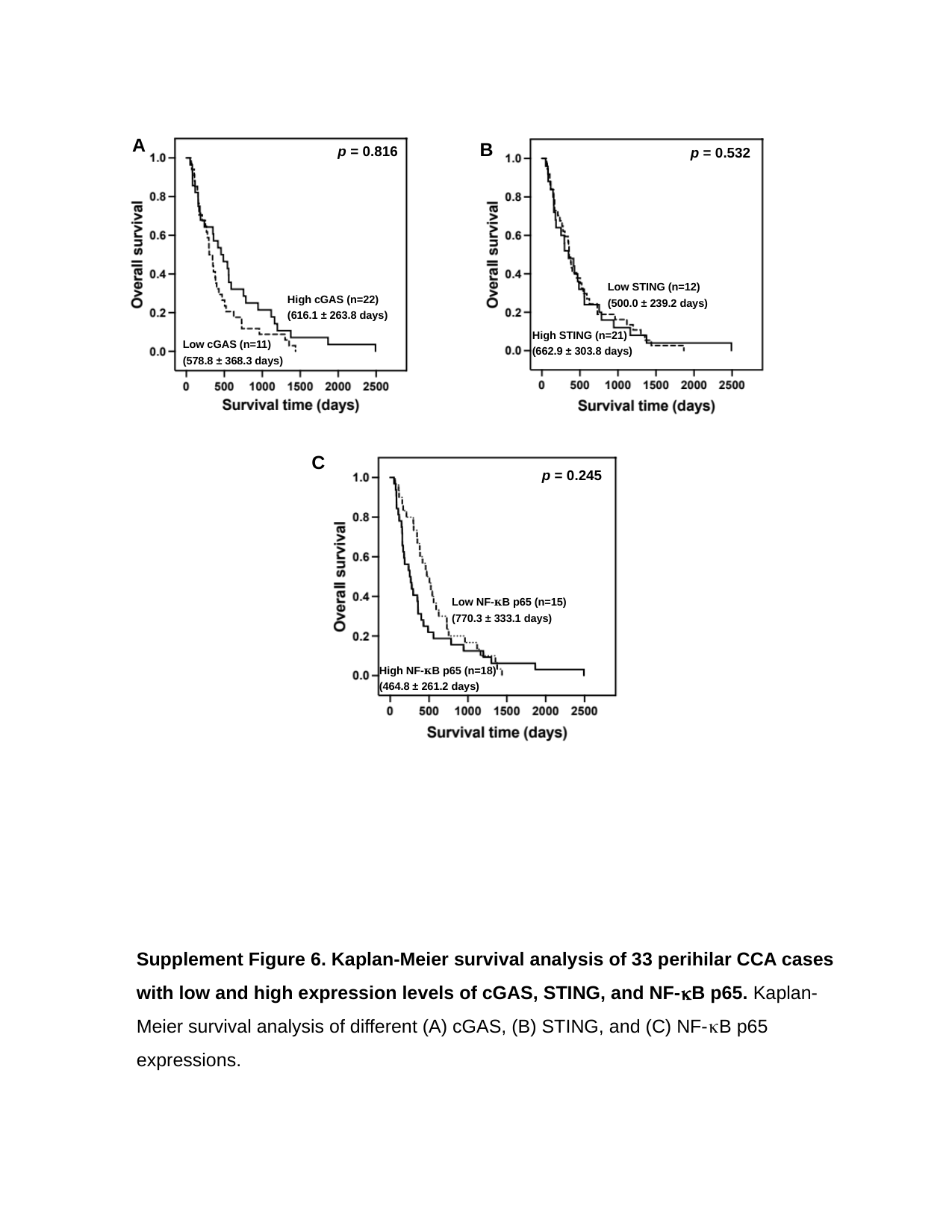

p = 0.816
High cGAS (n=22)
(616.1 ± 263.8 days)
Low cGAS (n=11)
(578.8 ± 368.3 days)
p = 0.532
Low STING (n=12)
(500.0 ± 239.2 days)
High STING (n=21)
(662.9 ± 303.8 days)
A
B
C
p = 0.245
Low NF-𝛋B p65 (n=15)
(770.3 ± 333.1 days)
High NF-𝛋B p65 (n=18)
(464.8 ± 261.2 days)
Supplement Figure 6. Kaplan-Meier survival analysis of 33 perihilar CCA cases with low and high expression levels of cGAS, STING, and NF-κB p65. Kaplan-Meier survival analysis of different (A) cGAS, (B) STING, and (C) NF-κB p65 expressions.
