# Supplementary material for: High cyclic GMP-AMP synthase and stimulator of interferon genes in cholangiocarcinoma suggest their potential as targets for treatment
Source: PeerJ. 2025 Aug 6;13:e19800. doi: 10.7717/peerj.19800 (PMC12335239; doi:10.7717/peerj.19800)
Supplement: Supplemental Information 2 — Correlations and Multivariate analysis using Cox regression. [file peerj-13-19800-s002.docx]

**Supplement Table 1.** **Correlations of cGAS, STING and** **NF-κB p65 expression levels with clinicopathological characteristics of iCCA patients**

| **Variables** | **n (%)** | **cGAS** | | ***p^#^*** | **STING** | | ***p^#^*** | **NF-κB p65** | | ***p^#^*** |
| --- | --- | --- | --- | --- | --- | --- | --- | --- | --- | --- |
|  |  | **Low** | **High** |  | **Low** | **High** |  | **Low** | **High** |  |
| **Age (year)** | | | | | | | | | | |
| < 56 | 29 (46.8) | 17 | 12 | 0.575 | 18 | 11 | 0.719 | 15 | 14 | 0.622 |
| ≥ 56 | 33 (53.2) | 17 | 16 |  | 19 | 14 |  | 15 | 18 |  |
| **Gender** | | | | | | | | | | |
| Male | 39 (62.9) | 23 | 16 | 0.394 | 26 | 13 | 0.144 | 18 | 21 | 0.647 |
| Female | 23 (37.1) | 11 | 12 |  | 11 | 12 |  | 12 | 11 |  |
| **Histological type** | | | | | | | | | | |
| Papillary | 27 (43.5) | 14 | 13 | 0.678 | 14 | 13 | 0.270 | 11 | 16 | 0.290 |
| Non-papillary | 35 (56.5) | 20 | 15 |  | 23 | 12 |  | 19 | 16 |  |
| **Tumor stage (7^th^ AJCC^##^)** | | | | | | | | | | |
| I-III | 28 (45.2) | 13 | 15 | 0.227 | 16 | 12 | 0.712 | 14 | 14 | 0.818 |
| IV | 34 (54.8) | 21 | 13 |  | 21 | 13 |  | 16 | 18 |  |
| **Tumor size** (n=57^✝^) | | | | | | | | | | |
| < 7 cm | 23 (40.4) | 16 | 7 | 0.035* | 16 | 7 | 0.142 | 15 | 8 | 0.026* |
| ≥ 7 cm | 34 (59.6) | 14 | 20 |  | 17 | 17 |  | 12 | 22 |  |

**Note.**

^#^*p-*values were determined by Pearson Chi-square test.

^##^Tumor stage is classified according to the 7^th^ AJCC (Edge & Compton 2010).

**p* < 0.05.

^✝^incomplete information.

**Supplementary Table 2.** **Multivariate analysis using Cox regression of iCCA patients’ clinicopathological characteristics**

| **Variables** |  | **n** | **HR** | **95%CI** | ***p*** |
| --- | --- | --- | --- | --- | --- |
| Age (years) | < 56 | 29 | 1 |  |  |
|  | ≥ 56 | 33 | 1.146 | (0.676-1.944) | 0.613 |
| Gender | Male | 39 | 1 |  |  |
|  | Female | 23 | 1.093 | (0.617-1.934) | 0.761 |
| Histological type | Papillary | 27 | 1 |  |  |
|  | Non-papillary | 35 | 1.996 | (1.105-3.606) | 0.022* |
| Tumor stage | I-III | 28 | 1 |  |  |
|  | IV | 34 | 0.861 | (0.493-1.505) | 0.600 |
| cGAS expression | < 300 | 34 | 1 |  |  |
|  | = 300 | 28 | 0.490 | (0.255-0.940) | 0.032* |
| STING expression | < 300 | 37 | 1 |  |  |
|  | = 300 | 25 | 1.275 | (0.701-2.317) | 0.426 |
| NF-κB p65 expression | <145  ≥ 145 | 30  32 | 1  2.180 | (1.180-4.028) | 0.013* |

**Note.**

HR, Hazard ratio; CI, Confidence interval; **p* < 0.05.

**Supplement Table 3.** **Correlations of cGAS, STING and** **NF-κB p65 expression levels with clinicopathological characteristics of pCCA patients**

| **Variables** | **n (%)** | **cGAS** | | ***p^#^*** | **STING** | | ***p^#^*** | **NF-κB p65** | | ***p^#^*** |
| --- | --- | --- | --- | --- | --- | --- | --- | --- | --- | --- |
|  |  | **Low** | **High** |  | **Low** | **High** |  | **Low** | **High** |  |
| **Age (year)** | | | | | | | | | | |
| < 56 | 18 (54.5) | 5 | 13 | 0.458 | 6 | 12 | 0.692 | 7 | 11 | 0.407 |
| ≥ 56 | 15 (45.5) | 6 | 9 |  | 6 | 9 |  | 8 | 7 |  |
| **Gender** | | | | | | | | | | |
| Male | 23 (69.7) | 7 | 16 | 0.592 | 8 | 15 | 0.775 | 11 | 12 | 0.678 |
| Female | 10 (30.3) | 4 | 6 |  | 4 | 6 |  | 4 | 6 |  |
| **Histological type** | | | | | | | | | | |
| Papillary | 16 (48.5) | 5 | 11 | 0.805 | 7 | 9 | 0.392 | 8 | 8 | 0.611 |
| Non-papillary | 17 (51.5) | 6 | 11 |  | 5 | 12 |  | 7 | 10 |  |
| **Tumor stage (7^th^ AJCC^##^)** | | | | | | | | | | |
| I-III | 15 (45.5) | 6 | 9 | 0.458 | 6 | 9 | 0.692 | 8 | 7 | 0.407 |
| IV | 18 (54.5) | 5 | 13 |  | 6 | 12 |  | 7 | 11 |  |
| **Tumor size** (n=21^✝^) | | | | | | | | | | |
| < 7 cm | 14 (66.7) | 4 | 10 | 0.204 | 3 | 11 | 0.306 | 6 | 8 | 0.537 |
| ≥ 7 cm | 7 (33.3) | 4 | 3 |  | 3 | 4 |  | 4 | 3 |  |

**Note.**

^#^*p-*values were determined by Pearson Chi-square test.

^##^Tumor stage is classified according to the 7^th^ AJCC (Edge & Compton 2010).

**p* < 0.05.

^✝^incomplete information.

**Supplementary Table 4.** **Multivariate analysis using Cox regression of pCCA patients’ clinicopathological characteristics**

| **Variables** |  | **n** | **HR** | **95%CI** | ***p*** |
| --- | --- | --- | --- | --- | --- |
| Age (years) | < 56 | 18 | 1 |  |  |
|  | ≥ 56 | 15 | 1.654 | (0.730-3.749) | 0.228 |
| Gender | Male | 23 | 1 |  |  |
|  | Female | 10 | 0.924 | (0.360-2.367) | 0.869 |
| Histological type | Papillary | 16 | 1 |  |  |
|  | Non-papillary | 17 | 0.820 | (0.347-1.937) | 0.651 |
| Tumor stage | I-III | 15 | 1 |  |  |
|  | IV | 18 | 5.353 | (1.669-17.168) | 0.005* |
| cGAS expression | < 300 | 11 | 1 |  |  |
|  | = 300 | 22 | 0.752 | (0.314-1.798) | 0.521 |
| STING expression | < 300 | 12 | 1 |  |  |
|  | = 300 | 21 | 0.654 | (0.301-1.422) | 0.284 |
| NF-κB p65 expression | <177  ≥ 177 | 15  18 | 1  1.263 | (0.550-2.899) | 0.582 |

**Note.**

HR, Hazard ratio; CI, Confidence interval; **p* < 0.05.
