# Supplementary figures and images for: High cyclic GMP-AMP synthase and stimulator of interferon genes in cholangiocarcinoma suggest their potential as targets for treatment
Source: PeerJ. 2025 Aug 6;13:e19800. doi: 10.7717/peerj.19800 (PMC12335239; doi:10.7717/peerj.19800)

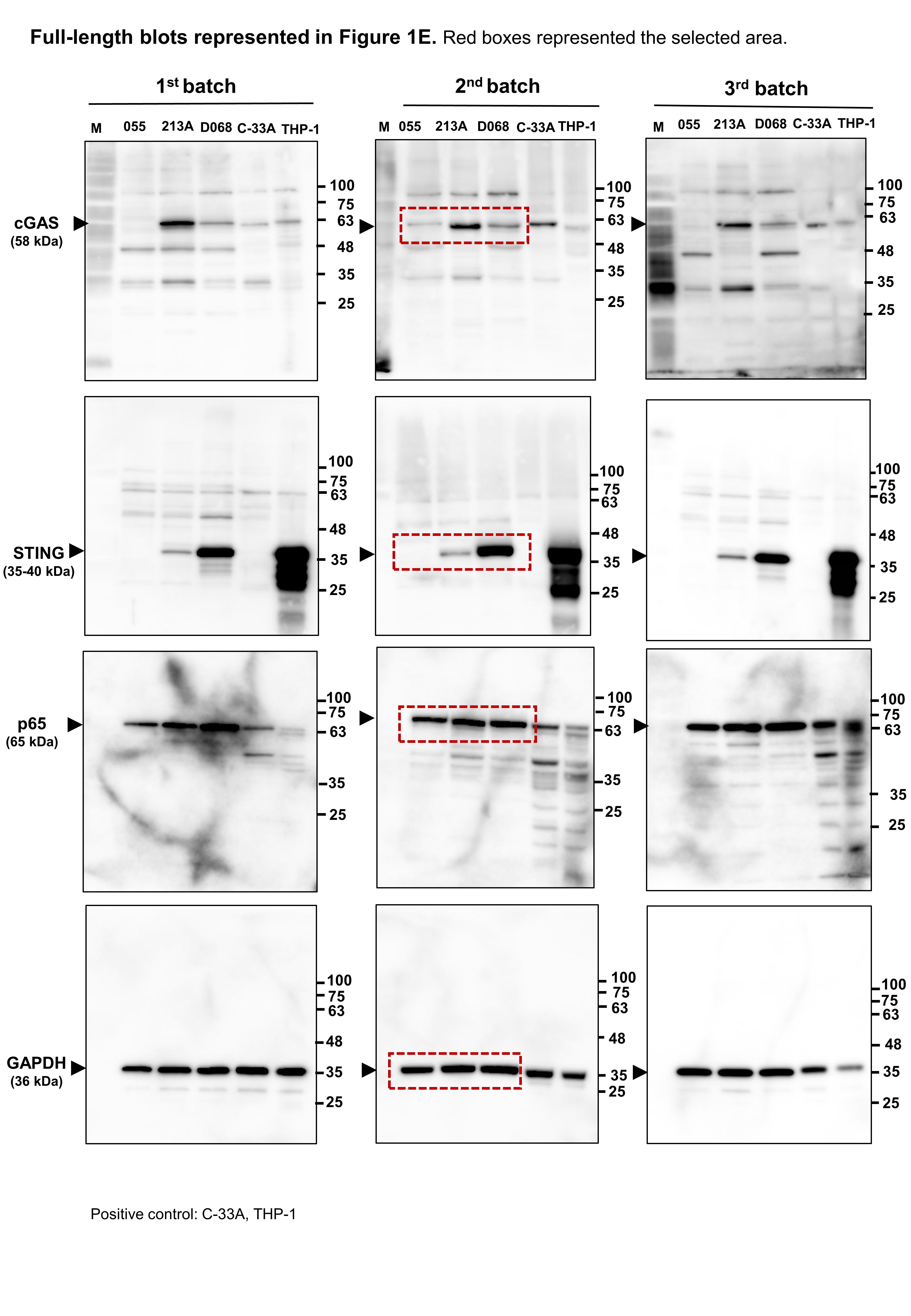

Supplement: Supplemental Information 3 [file peerj-13-19800-s003.jpg]

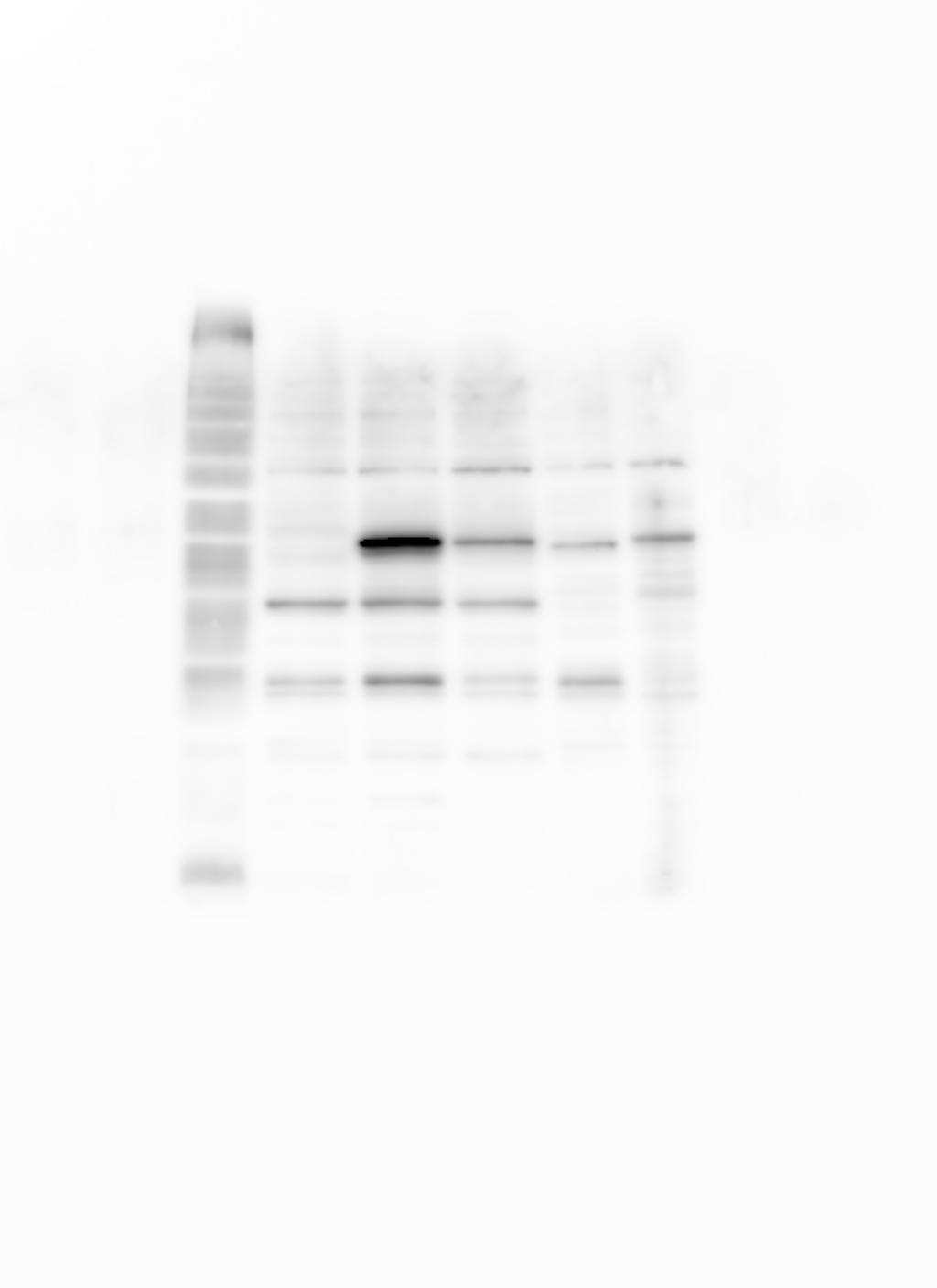

Supplement: Supplemental Information 5 [file peerj-13-19800-s005.zip › Raw_data/1st_batch_cGAS.jpg]

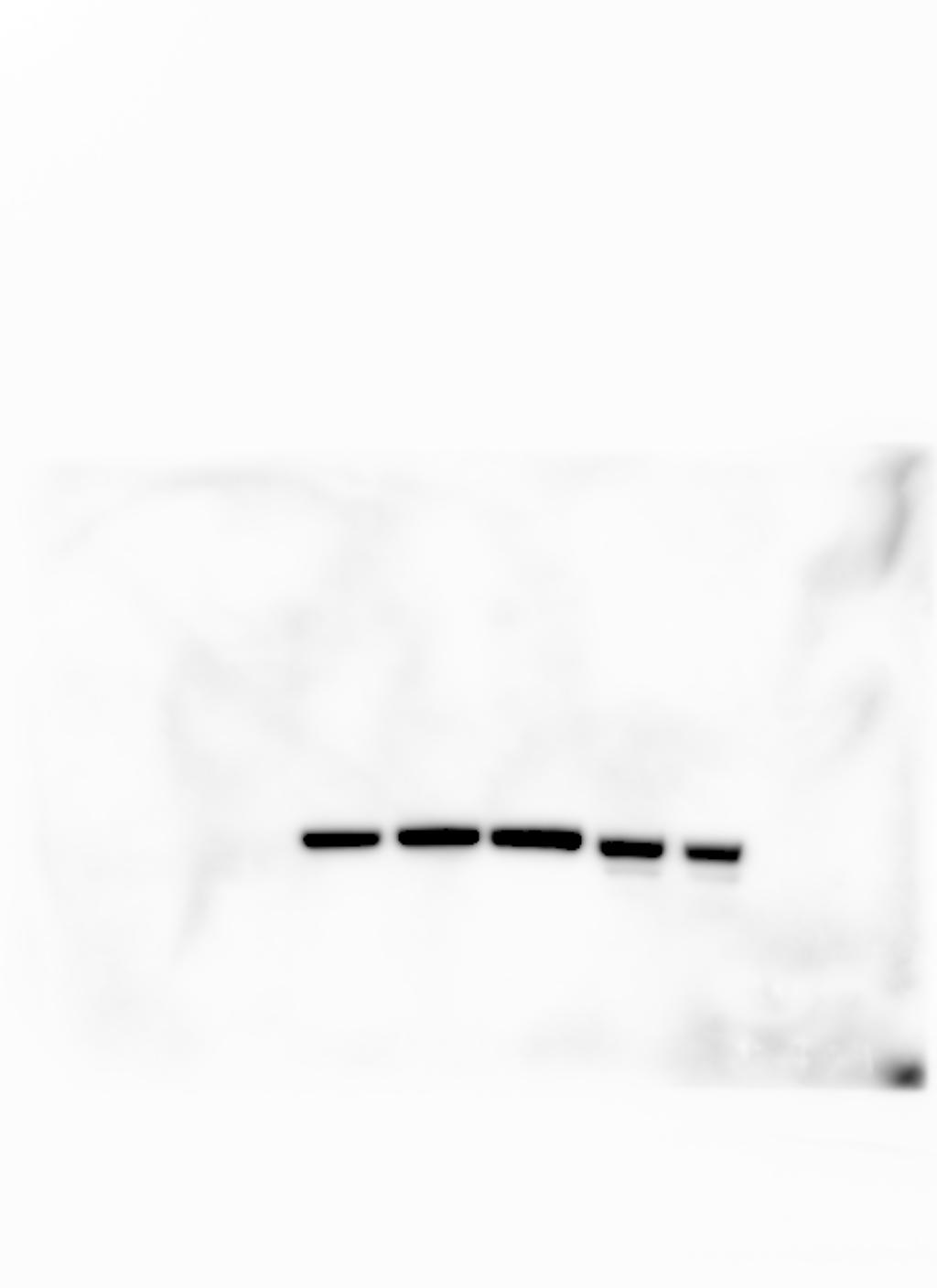

Supplement: Supplemental Information 5 [file peerj-13-19800-s005.zip › Raw_data/2nd_batch_GAPDH.jpg]

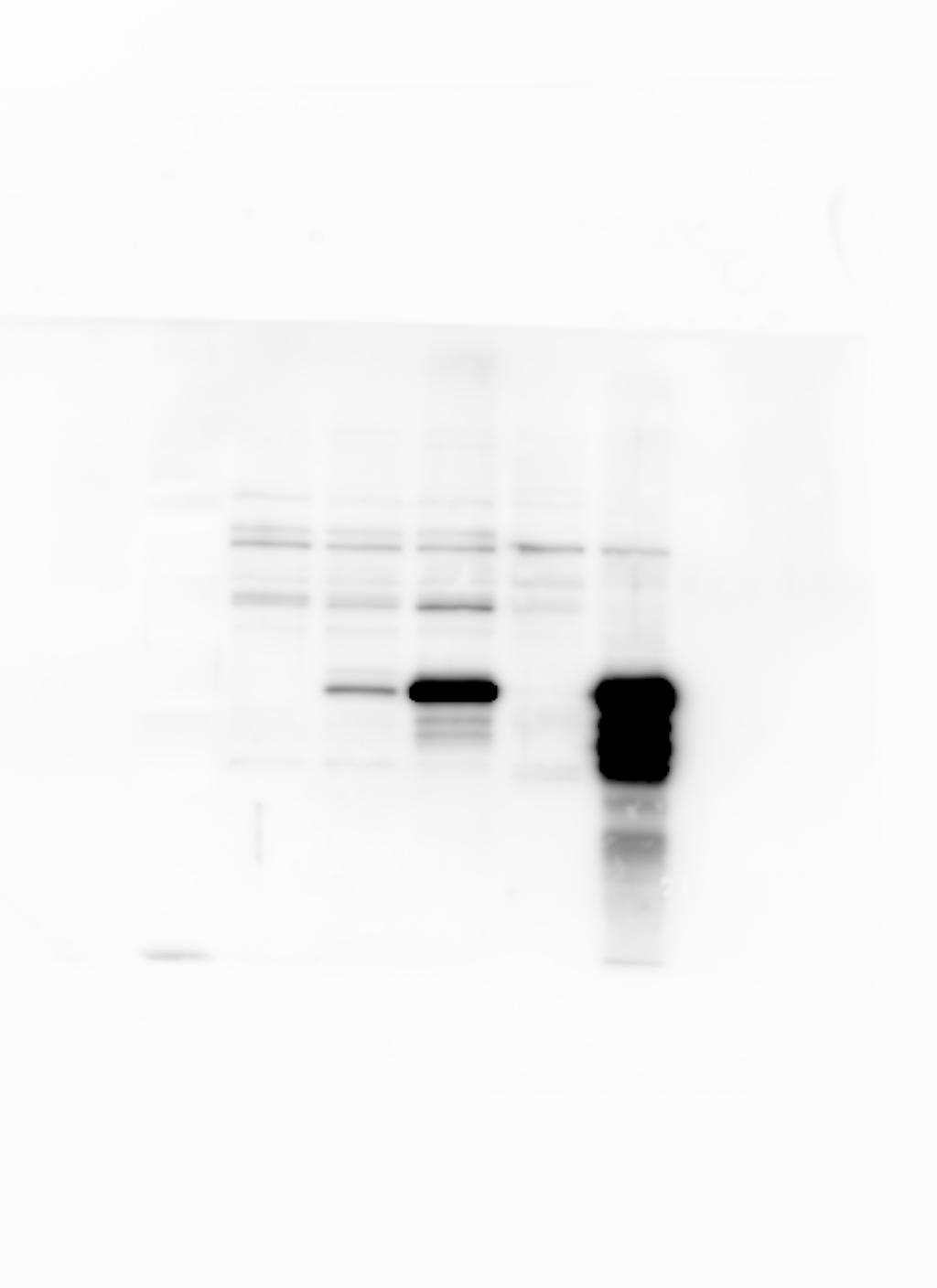

Supplement: Supplemental Information 5 [file peerj-13-19800-s005.zip › Raw_data/1st_batch_STING.jpg]

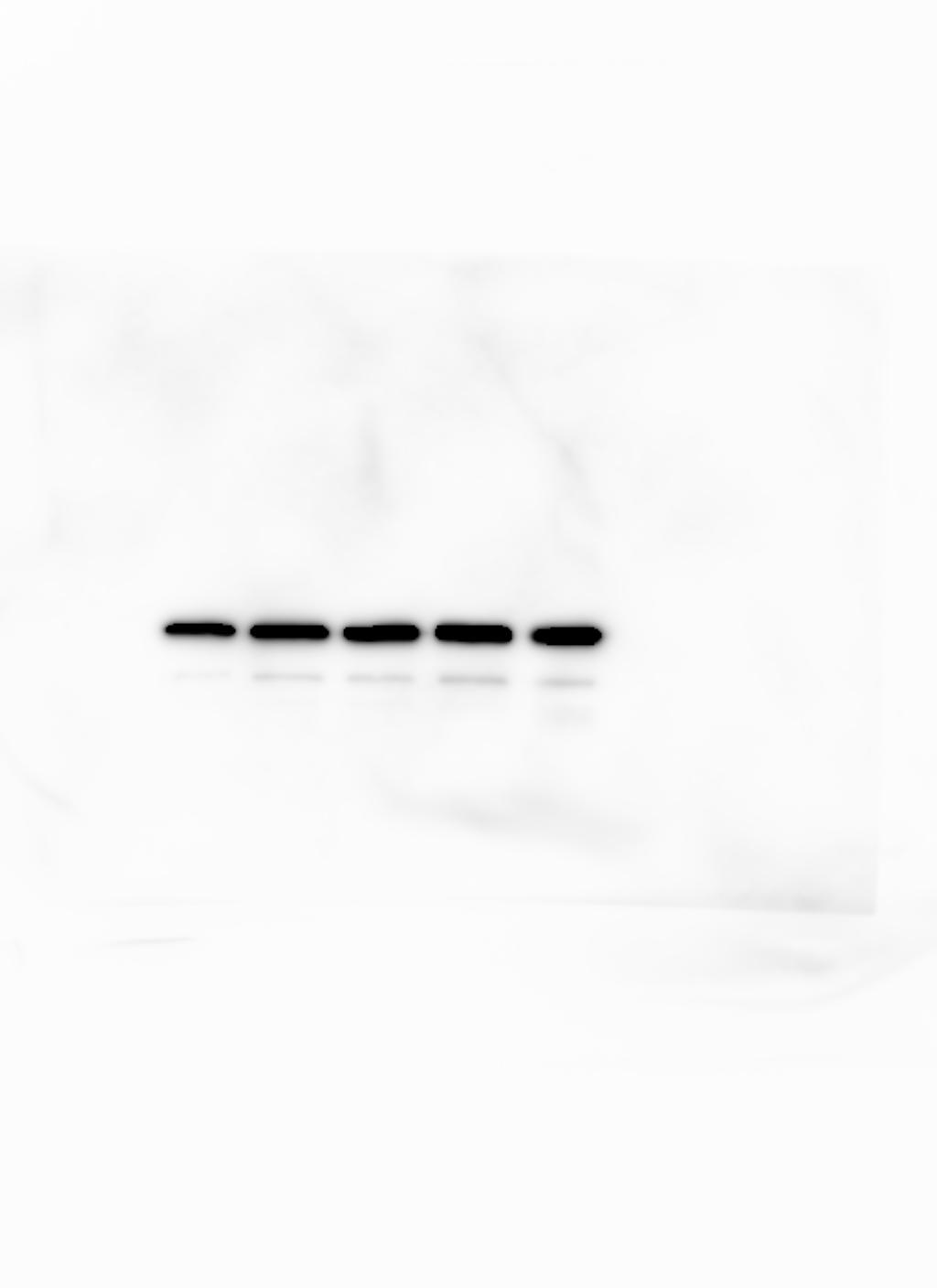

Supplement: Supplemental Information 5 [file peerj-13-19800-s005.zip › Raw_data/1st_batch_GAPDH.jpg]

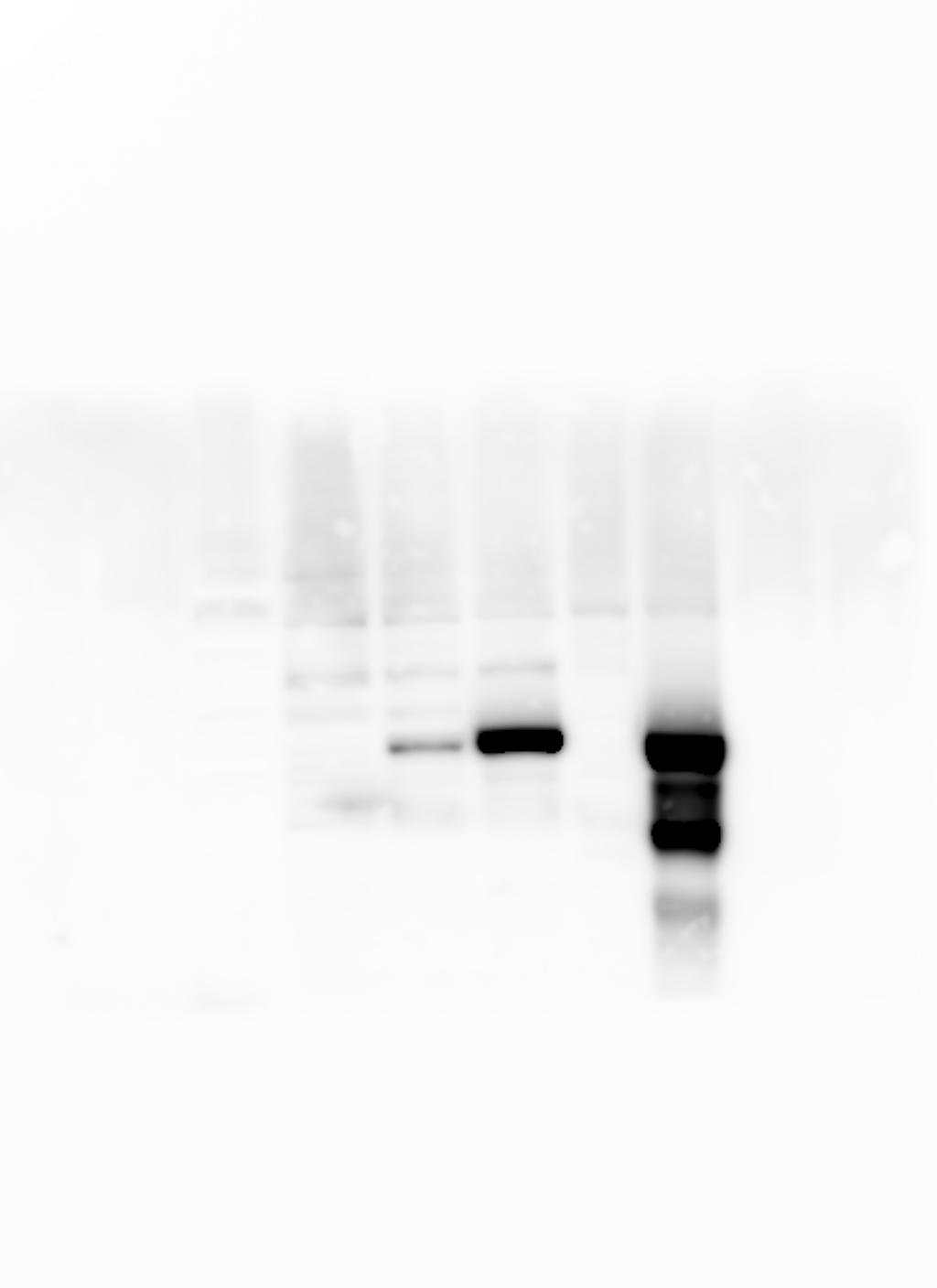

Supplement: Supplemental Information 5 [file peerj-13-19800-s005.zip › Raw_data/2nd_batch_STING.jpg]

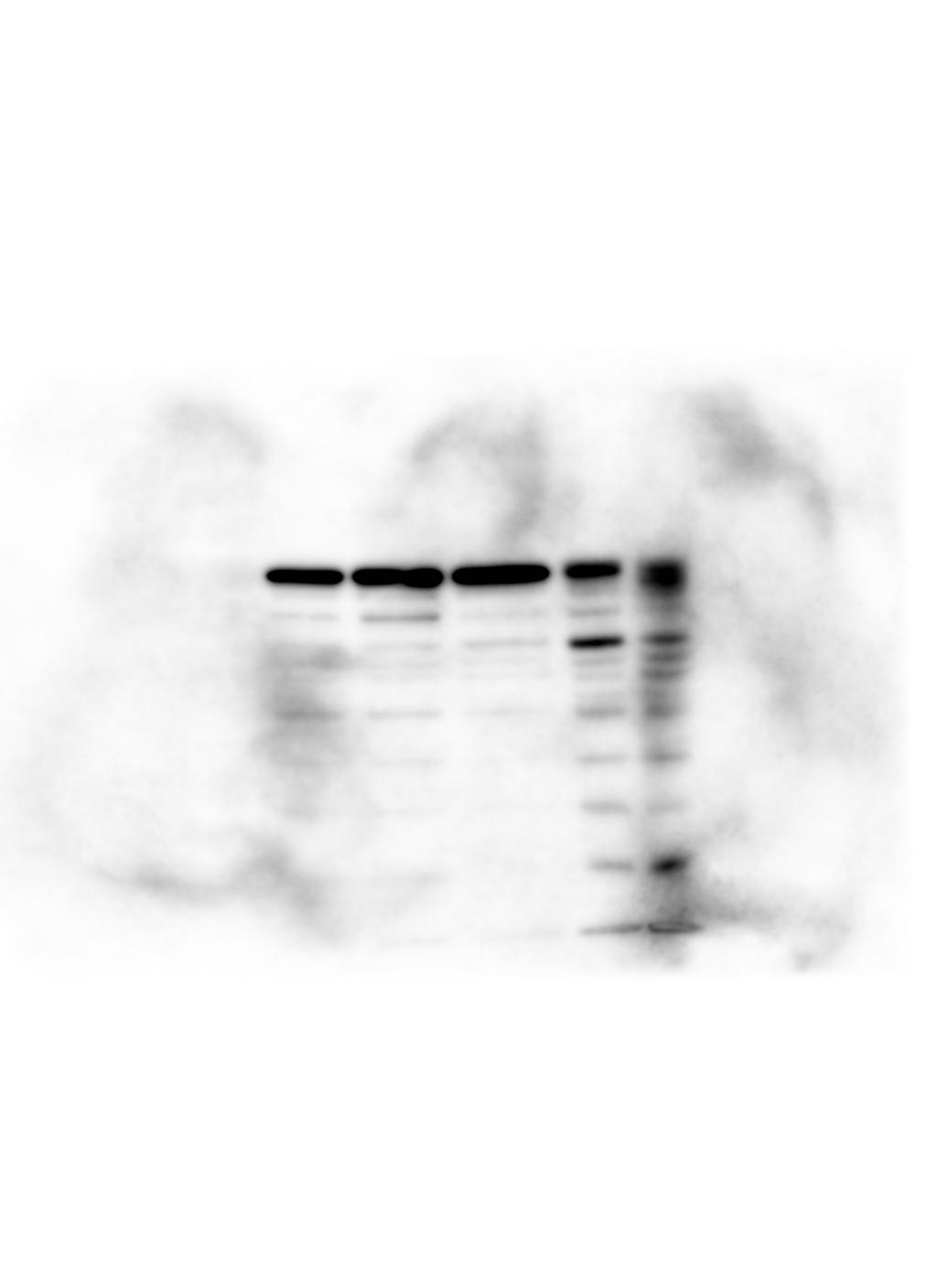

Supplement: Supplemental Information 5 [file peerj-13-19800-s005.zip › Raw_data/3rd_batch_NF-kB p65.jpg]

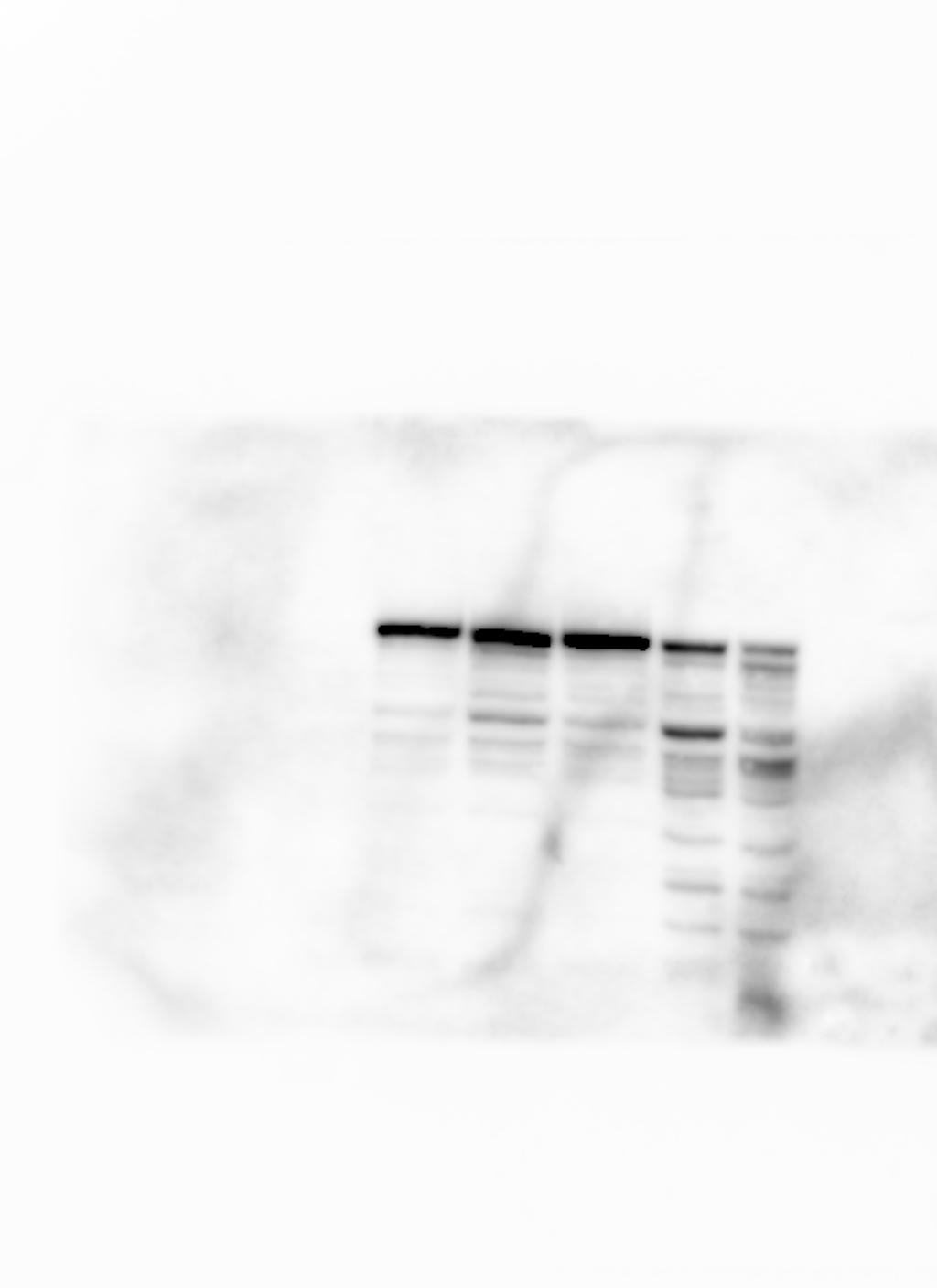

Supplement: Supplemental Information 5 [file peerj-13-19800-s005.zip › Raw_data/2nd_batch_NF-kB p65.jpg]

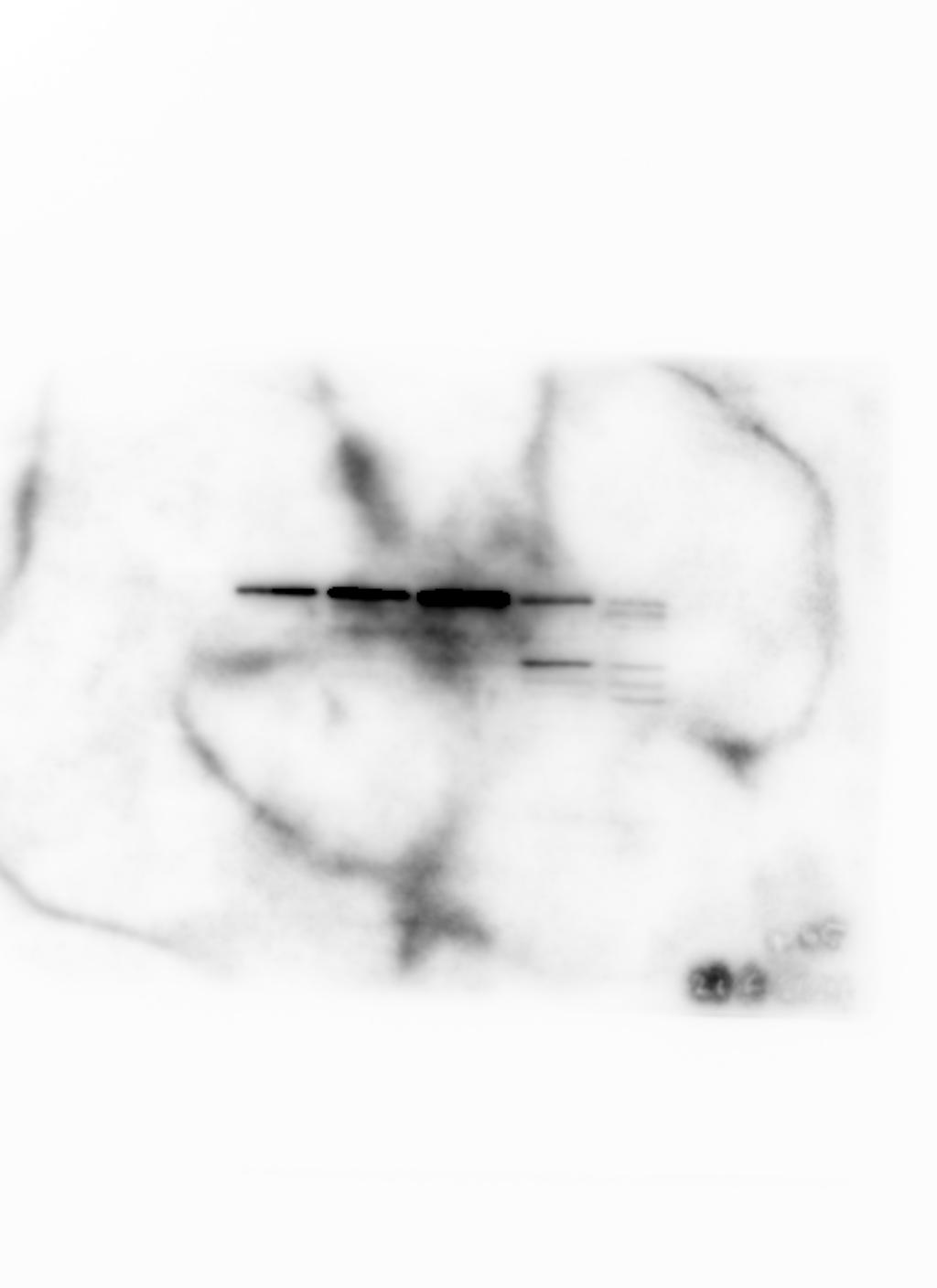

Supplement: Supplemental Information 5 [file peerj-13-19800-s005.zip › Raw_data/1st_batch_NF-kB p65.jpg]

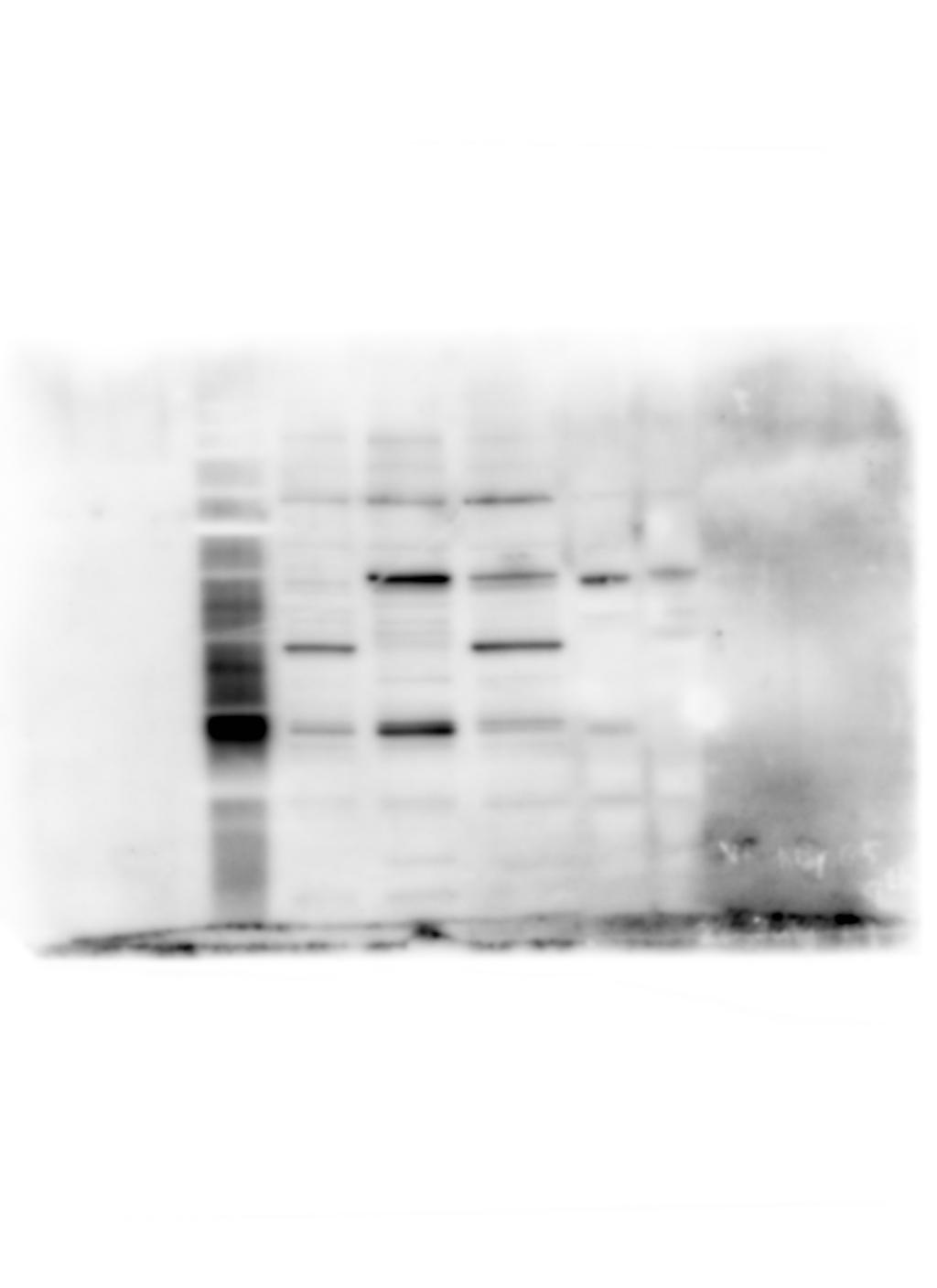

Supplement: Supplemental Information 5 [file peerj-13-19800-s005.zip › Raw_data/3rd_batch_cGAS.jpg]

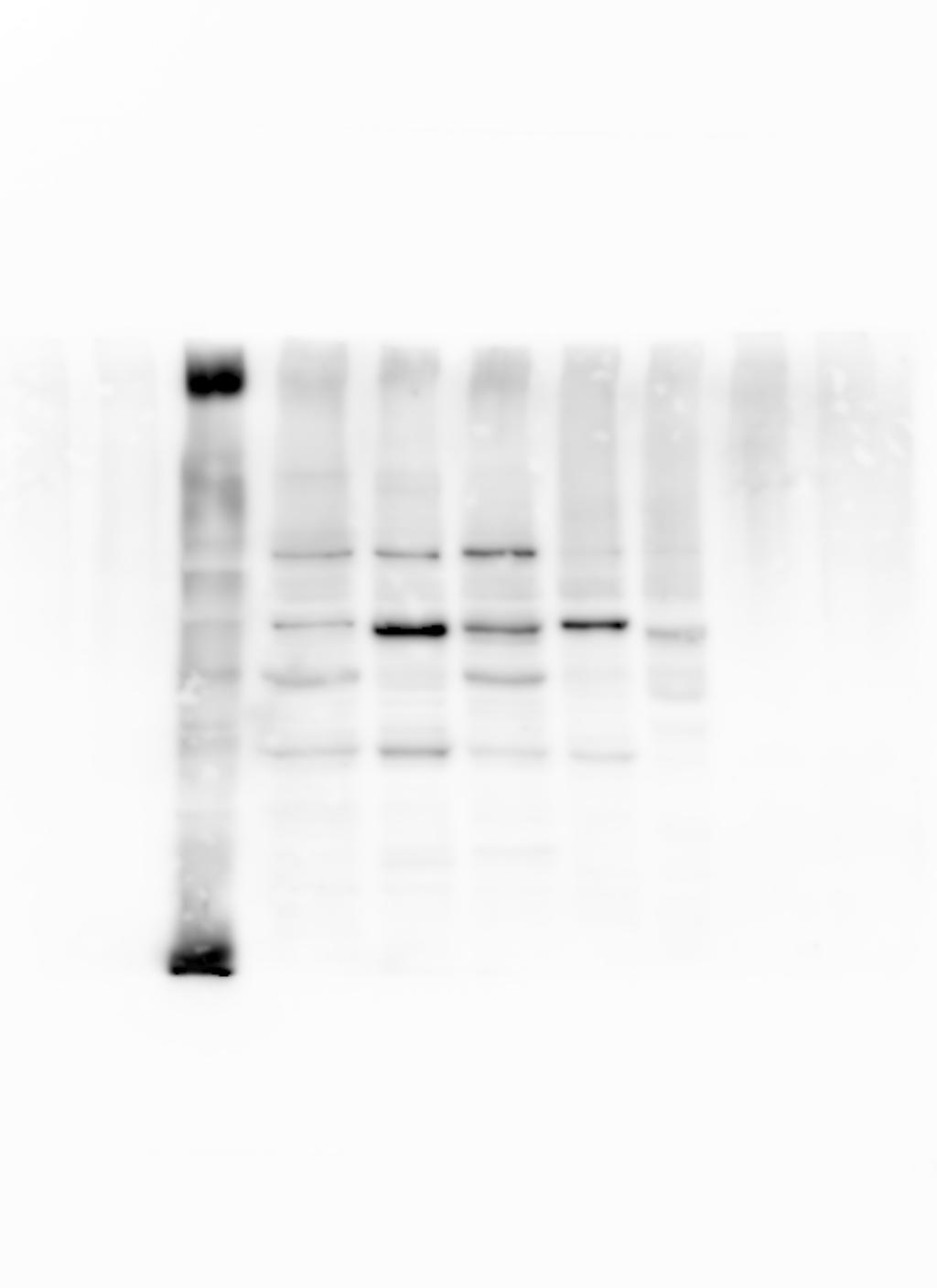

Supplement: Supplemental Information 5 [file peerj-13-19800-s005.zip › Raw_data/2nd_batch_cGAS.jpg]

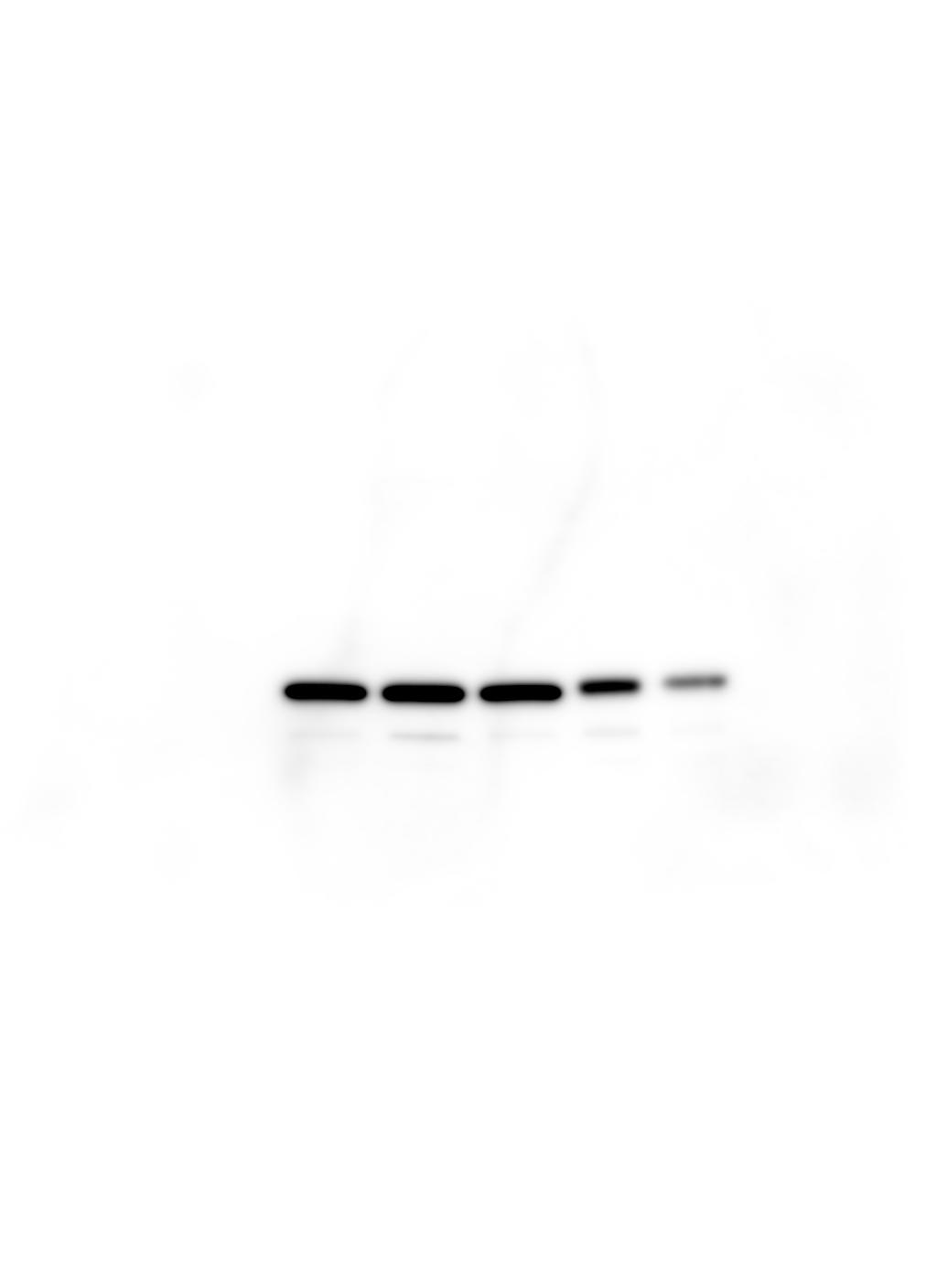

Supplement: Supplemental Information 5 [file peerj-13-19800-s005.zip › Raw_data/3rd_batch_GAPDH.jpg]

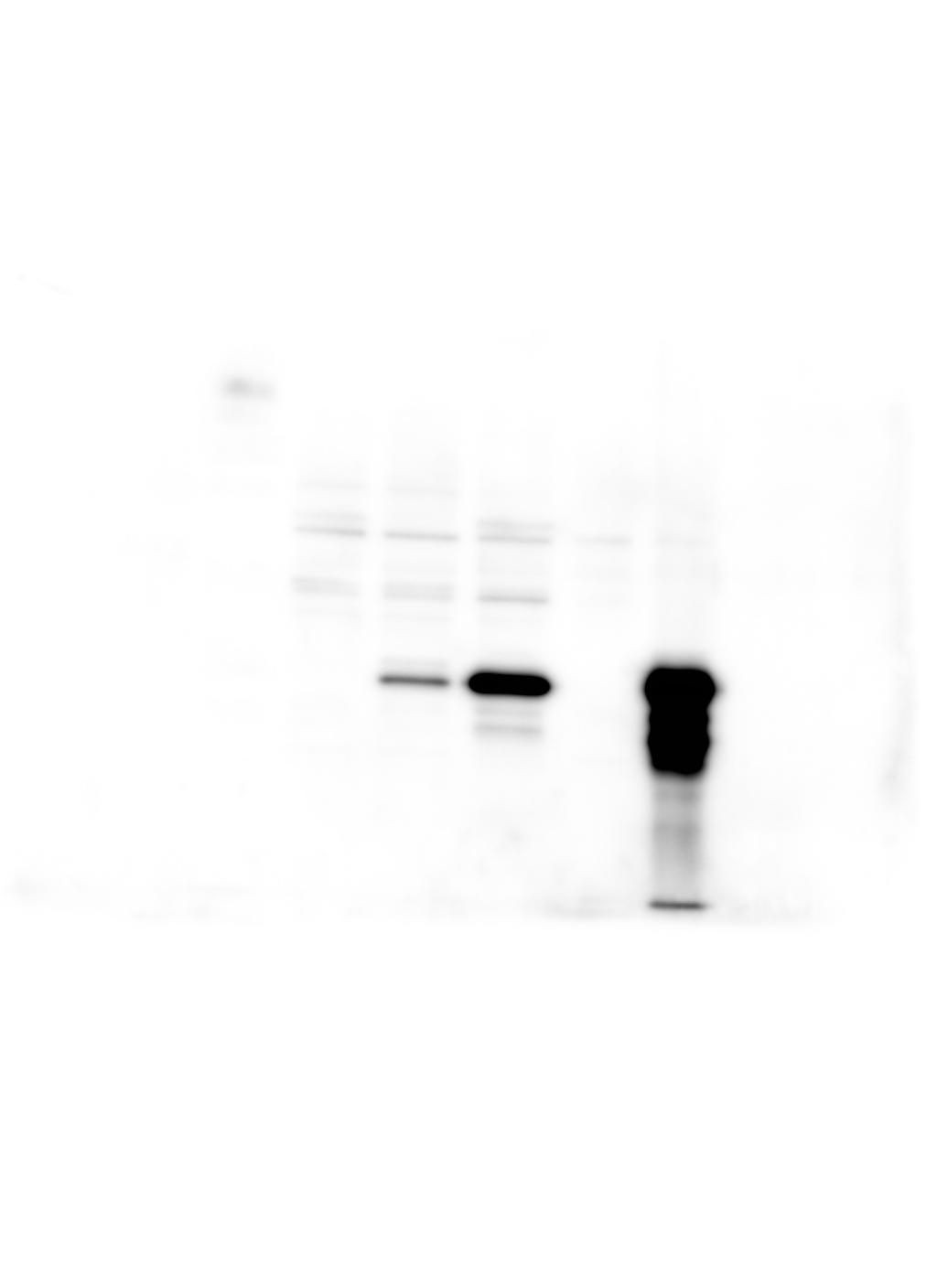

Supplement: Supplemental Information 5 [file peerj-13-19800-s005.zip › Raw_data/3rd_batch_STING.jpg]
